# Combination of marine bioactive compounds and extracts for the prevention and treatment of chronic diseases

**DOI:** 10.3389/fnut.2022.1047026

**Published:** 2023-01-12

**Authors:** Belgheis Ebrahimi, Saeid Baroutian, Jinyao Li, Baohong Zhang, Tianlei Ying, Jun Lu

**Affiliations:** ^1^School of Science, Faculty of Health and Environmental Sciences, Auckland University of Technology, Auckland, New Zealand; ^2^Department of Chemical and Materials Engineering, University of Auckland, Auckland, New Zealand; ^3^Xinjiang Key Laboratory of Biological Resources and Genetic Engineering, College of Life Science and Technology, Xinjiang University, Xinjiang, China; ^4^School of Pharmacy, Shanghai Jiao Tong University, Shanghai, China; ^5^Key Laboratory of Medical Molecular Virology of MOE/MOH, Shanghai Medical College, Fudan University, Shanghai, China; ^6^Auckland Bioengineering Institute, University of Auckland, Auckland, New Zealand; ^7^Institute of Biomedical Technology, Auckland University of Technology, Auckland, New Zealand; ^8^Maurice Wilkins Centre for Molecular Discovery, Auckland, New Zealand; ^9^College of Life Sciences and Oceanography, Shenzhen University, Shenzhen, Guangdong, China; ^10^College of Food Engineering and Nutrition Sciences, Shaanxi Normal University, Xi'an, Shaanxi, China; ^11^College of Food Science and Technology, Nanchang University, Nanchang, Jiangxi, China

**Keywords:** marine bioactives, marine nutraceutical combination, antioxidant, anti-inflammatory, anticarcinogenic, anti-obesity

## Abstract

**Background:**

In recent years, marine-based functional foods and combination therapy are receiving greater recognition for their roles in healthy lifestyle applications and are being investigated as viable and effective strategies for disease treatment or prevention.

**Aim of the review:**

This review article presents and discusses the relevant scientific publications that have studied the synergistic and additive effects of natural marine bioactive compounds and extract combinations with anti-obesity, anti-inflammatory, antioxidant, and chemopreventive activities in the last two decades. The paper presents the mechanism of action and health benefits of developed combinations and discusses the limitation of the studies. Furthermore, it recommends alternatives and directions for future studies. Finally, it highlights the factors for developing novel combinations of marine bioactive compounds.

**Key scientific concepts of review:**

Combination of marine bioactive compounds or extracts affords synergistic or additive effects by multiple means, such as multi-target effects, enhancing the bioavailability, boosting the bioactivity, and neutralizing adverse effects of compounds in the mixture. For the development of marine-based combinations, there are key points for consideration and issues to address: knowledge of the mechanism of action of individual compounds and their combinations, optimum ratio and dosing of compounds, and experimental models must all be taken into account. Strategies to increase the number and diversity of marine combinations, and further development of marine-based functional foods, are available. However, only a small number of natural marine bioactive combinations have been assessed, and most research has been focused on fish oil and carotenoid synergy. Therefore, more research and resources should be spent on developing novel marine bioactive combinations as functional foods and nutraceuticals.

## Introduction

Chronic non-communicable diseases, such as obesity, diabetes, arthritis, cancer, and cardiovascular diseases, are increasing worldwide. The increase is mainly linked to changes in dietary habits. Increased awareness of the link between food intake habits and health has led many people to seek functional food options in their meal plans. Functional foods are defined as foods with additional or improved benefits beyond the normal nutritional value. This additional value could reduce the risk of chronic diseases. Nutraceuticals may also be extracted from foods to develop capsules or tablets as accessible supplements to dietary intake. The food ingredient market is rapidly growing, and a new source of bioactive substances is needed for functional food industries (1, 2).

Marine microbiota and fauna are valuable sources of bioactive compounds for the food and pharmaceutical industries. Bioactive compounds can be isolated from macroalgae (seaweeds), microalgae, echinoderms, crustaceans (for example, crayfish, crab, shrimp, and lobster), cephalopods (such as squid, cuttlefish, and octopus), mollusks (including mussel, clam, oyster, scallop, abalone, snail, and conch) and fish (3). So far, more than 36,000 compounds have been isolated from marine micro-and macro-organisms. The most widely isolated and researched marine bioactive compounds include carbohydrates, pigments, polyphenols, peptides, proteins, essential fatty acids, vitamins, and minerals. Marine compounds have antioxidant, anti-thrombotic, anti-coagulant, anti-inflammatory, anti-proliferative, anti-hypertensive, anti-diabetic, and cardio-protective activity; therefore, they can be used as active ingredients in functional food, nutraceuticals, dietary supplements, prebiotics, and pharmaceuticals (3, 4). Significantly, such bioactive compounds can minimize chronic non-communicable disease risk by reducing the onset of inflammation and oxidation (5).

Natural foods typically have low concentrations of nutraceuticals. As a result, large quantities of food need to be ingested to achieve the desired results in the human body (6). However, excessive consumption of certain bioactive compounds might have toxic effects or interact with medications (7). Additionally, when functional food is taken orally, most bioactive compounds are affected by the conditions of the gastrointestinal system, such as pH, metabolic enzymes, or normal microbiota of the gastrointestinal tract, which can reduce or eliminate their effectiveness or bioavailability (8). These issues can be solved by the concept of combinations which forwards the premise that disease prevention and/or treatment activities resulting from the combined impact of various compounds (influencing multiple mechanisms of action) are more successful than those caused by an individual compound that participates in only a single molecular mechanism. In addition, combinations of bioactive compounds can reduce the toxicity and side effects of the individual compounds (6).

**Figure 1:**
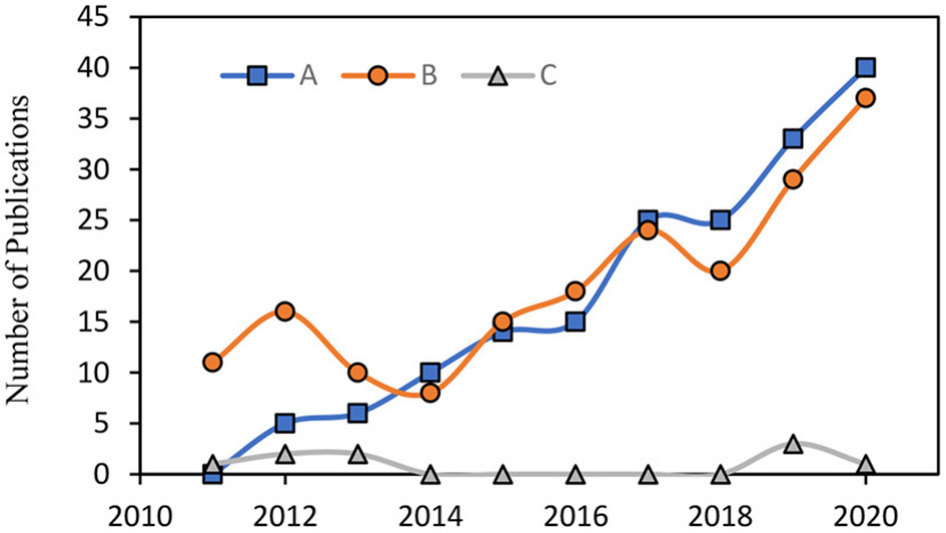
The number of publication featuring (A) “marine bioactive compounds” + “combination” in Scopus. (B) “marine bioactive compounds” + “combination” in PubMed. (C) “natural marine bioactive compounds” + “combination” in Scopus & PubMed & Google Scholar.

The number of publications on the combination of marine bioactive compounds has increased in the last decade. These combinations include two main categories: “bioactive compound-drug combinations” and “natural bioactive compound combinations.” Overall, 26 keyword combinations were used (a total of 4,384 articles were screened), and out of 4,384 articles, 29 articles were included in this manuscript. The rest of the articles were found by citations or by reading the suggested related articles. In the first category, marine bioactive compounds are combined with pharmaceuticals to support drug therapy (9). In contrast, in the second category, the combinations are composed of only naturally occurring bioactive compounds. Most studies focus on the first category. Although marine bioactive compounds have received high interest due to their therapeutic capability, studies on the second category remain sparse (Figure 1).

Over the last two decades, several review articles have investigated different combinations of phytochemicals and plant extracts on outcomes of disease prevention or treatment (10–17). To our knowledge, no review article has discussed and summarized the natural bioactive compound combinations. The main objective of this review article is to provide an overview of the scientific evidence published in the last two decades on the interaction effects of combinations of marine bioactive compounds and extracts on the treatment and prevention of various chronic non-communicable diseases. The studies related to bioactive compound-drug combinations and drug therapy are not included in this paper. The first part of this review canvasses the general mechanisms of interaction and the advantages of the combination strategy. The second part of the review discusses the health benefit of the combination of marine bioactive compounds and extracts for preventing or treating chronic diseases and elaborates on types and mechanisms of interaction. The final part of the review examines the factors for developing new combinations of marine bioactive compounds and the future research direction in the specialty.

## Underlying mechanisms of interaction and assessing combination effects

The biological effects of bioactive compound mixtures can be higher or lower than the summative effects of each component. The interactions between multiple agents are classified as potentiation, addition, synergy, or antagonism. In potentiation interaction, the combination of the two active and non-active compounds has a more significant effect than that of the single active ingredient, where the involvement of the inactive compound improves the efficacy of the active compound (18). Some natural compounds may not possess specific effects themselves but increase the solubility, absorption, distribution, or metabolism *inappropriate concentration of bioactive compounds in*. Therefore, the combinations have beneficial effects by increasing the bioavailability of active compounds (16). If each component of the combination is active, it can produce an additive, synergistic or antagonistic effect. In additive interactions, the overall effect is equal to the sum efficacy of single components of the combination. If the combined effect is greater than adding individual compounds, the interaction is synergistic. However, if the combined effect is less than the addition of individual compounds, the interaction is considered antagonistic (18). A multitude of mechanisms can result in synergies, such as multi-target effects (pharmacodynamic synergism), modulation of drug transport, permeation, and bioavailability (pharmacokinetic synergism), and elimination of adverse effects of bioactive compounds (16).

The combination response observed in studies must be compared to an accepted reference model to determine the type of interaction. The most popular model is the combination index (CI) which is commonly used in both chemical- and cell-based studies. It was first established to evaluate the interactions between different drug combinations. Lately, CI has been used in food extracts and bioactive compound evaluation (17). The CI quantitatively calculates the combinatorial effect, and there is no limitation for the number of individual compounds in the combination. It is defined in the Equation as follows:


$$\begin{array}{l} \text{CI}=\frac{\text{d}1}{\text{Dx}1}+\;\frac{\text{d}2}{\text{Dx}2} \end{array}$$

**Figure 2:**
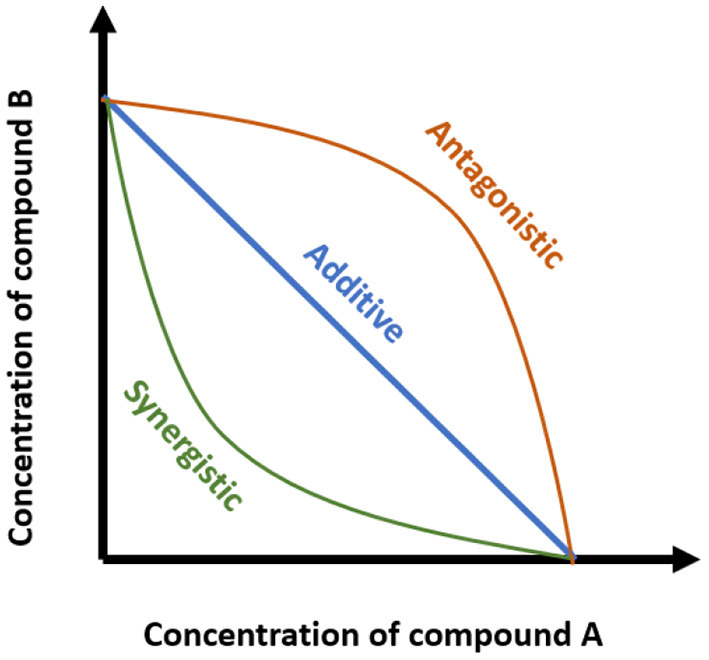
Isobole method showing different types of combination effects (synergistic, antagonistic, and additive). Axes represent the concentration of the individual compounds, and the points represent the combination of concentrations of the two compounds required to reach a particular fixed effect.

d1 and d2 are the respective combination doses of drug one and drug two that produce an effect x, and Dx1 and Dx2 are the corresponding single doses for drug one and drug two that result in the same effect x. If the CI is < 1, the interaction is a synergistic effect, while CI values higher than 1 indicate antagonism. A CI value equal to 1 is an additive effect. Another reference model is isobologram or Isobole method. It is a graphical procedure that assists in representing the trends of combination responses. The type of interaction depends on the position of the dose of combination on the “iso-effect” linear line (Figure 2) (19). However, this method is rarely used to evaluate bioactive compound interactions (17). Besides the CI and Isobole, other methods can be used to evaluate the combined effects, such as curve-shift analysis and universal surface response analysis. These two methods yield a statistical estimate of differentiation between synergy, additivity, and antagonism. In general, all four mentioned methods provide complementary information (20). In some studies, the result of the interaction is determined as synergistic or additive, but no method has been used to analyze the interaction mode. In this review, these outcomes are referred to as potential synergy or additive.

**Figure 3:**
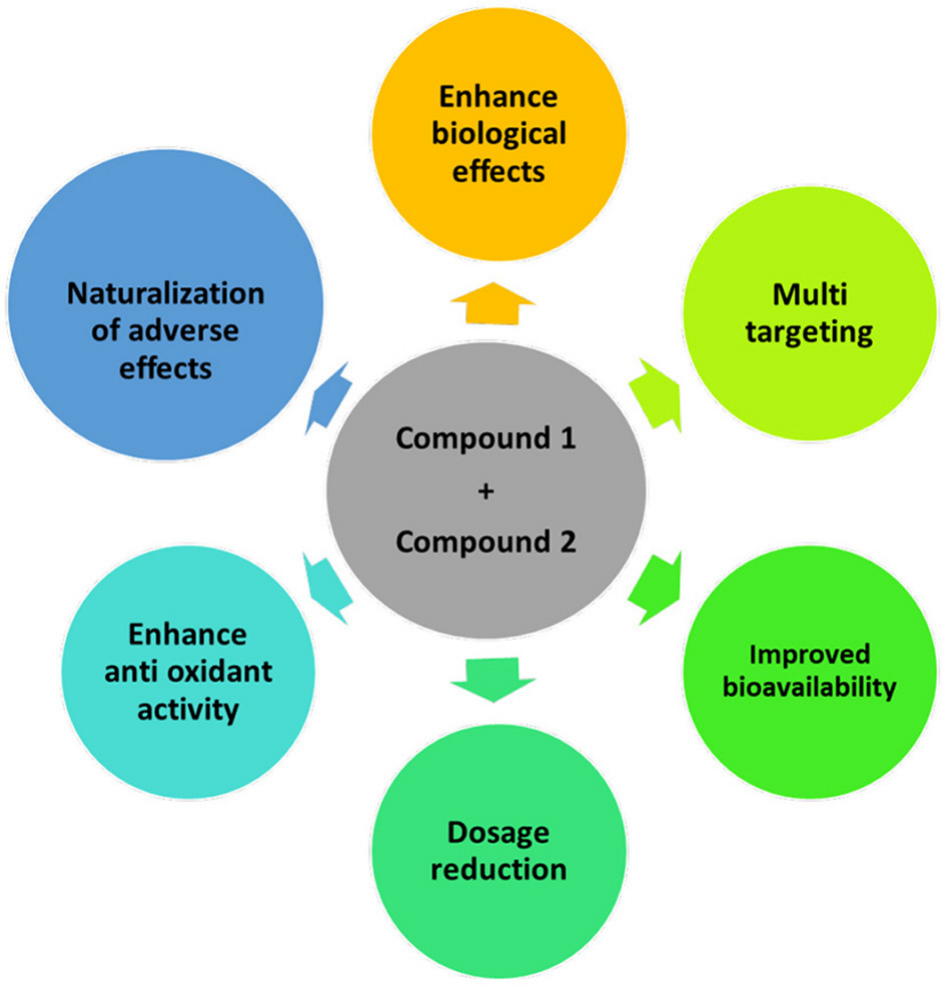
Mechanism of synergism—improvement in antioxidant activity, bioavailability, and biological effects of compounds; reduction of the dosage of individual compounds; act on new targets or multiple targets, or multiple pathways; naturalization of the adverse effect of individual compounds.

The mechanism of combination is complex and cannot be easily predicted from the activity of each compound in the mixture; therefore, understanding the synergistic mechanism is vital in selecting combinations. Moreover, the combination mechanism depends on several different variables, such as concentration, ratio, orientation, reaction medium, nature of radical initiators, interfering substances, and the microenvironment (21). For example, an inappropriate concentration of bioactive compounds in a mixture may lower its biological effects. Sulfated polysaccharides extracted from *Eisenia arborea* and *Solieria filiform* showed *in vitro* antiviral activity on the measles virus and low cytotoxicity at inhibitory concentrations. When the synergistic effect of the two compounds was studied, the result showed that the synergistic effect of the combination was observed at low concentrations of extracts (96% inhibition with 0.0274 μg/mL and 0.011 μg/mL of *E. arborea* and *S. filiformis* sulfated polysaccharides, respectively.) in comparison to the higher individual extract effects (50% inhibition with 0.275 and 0.985 μg/mL of *E. arborea* and *S. filiformis*, respectively). Conversely, results showed an antagonism effect at high concentrations of extracts (22). Alongside the concentration, the proportion or ratio of individual compounds also plays an essential role in the synergistic activity of the mixture. Different ratios of bioactive compounds in combination can affect the synergistic interaction. For instance, Todorova et al. based on the CI calculation reported that the taxifolin/fucoidan combination at a ratio of 1:3 (CI 0.55) has a greater synergistic effect than the observed lower effect at a 3:1 ratio (CI 0.80) (23). Overall, the mechanism of synergy can be determined by increasing the bioavailability, enhancing the antioxidant activity, inhibiting lipid peroxidation, neutralization of adverse effects, and developing multi-target effects in different positions of a similar signaling cascade (Figure 3) (21).

## Method

A literature search was conducted using the PubMed, Scopus, and Google Scholar databases, and the search terms “Marine Bioactive Compounds,” “Antioxidant Activity,” “Anti-Inflammatory Activity,” “Anticancer Activity,” “Synergistic Effect,” and “Additive Effect” were used. In this review, we focused on relevant articles and reviews on the health benefits of the combination of bioactive compounds and extracts from marine organisms for the prevention or treatment of chronic non-communicable diseases from 2000 to 2022. Studies on combinations of marine bioactive compounds with synthetic drugs and drug therapy are excluded. The results are presented and discussed in the following sections.

## Benefits of the combination of marine compounds

### Effects of bioactive compounds' interactions on anti-obesity activity

The prevalence of obesity has increased worldwide in the past few decades and is ascribed predominantly to poor eating habits. Many epidemiological studies show a decreased prevalence of obesity-related illnesses in those who consume seafood-rich diets, suggesting that marine foods have a positive effect against obesity. This disorder is not only limited to being overweight but can include comorbidities such as hyperlipidemia, cardiovascular diseases, type 2 diabetes, and non-alcoholic fatty liver (NAFLD). Many compounds have been extracted and tested on anti-obesity effects from marine organisms, mostly from algae. Additionally, the anti-obesity properties of lipids obtained from marine sources are well known (Table 1). Developing functional foods from marine bioactive compounds could be an opportunity for the food industry to prevent and/or treat obesity (32, 33).

**Table 1:** List of compounds isolated from marine sources with potential anti-obesity activity.

**Compound** **name/class**	**Marine source**	**Experimental method**	**Mechanism of action**	**References**
Fish oil	Fish	High-fat-fed rats	Unregulated UCP1 expression in BAT/Enhanced peroxisomal fatty acid oxidation	(24)
		High-fat-fed rat & Clinical trial	Suppressing VLDL synthesis & hepatic lipogenesis	(25, 26)
		High-fat-fed mice	Decrease in lipogenesis, & activated PPARα-mediated fat oxidation	(27)
Fucoidan/sulphated polysaccharide	Brown algae *Ecklonia cava*	3T3-L1 cells High-fat diet induced obese mice	Reduce white adipose tissue lipid accumulation and increasing energy expenditure	(28)
Fucosylated chondroitin sulfate/Glycosaminoglycan	*Acaudina molpadioides*	3T3-L1 cells	Enhanced expression of Wnt/β-catenin related factors, as well as PPARγ and SREBP1 expression	(29)
Astaxanthin/Carotenoid	*Haematococc-us pluvialis*	Otsuka long evans tokushima fatty rat	Reduce visceral adipose tissue and ameliorate lipid profile	(30)
Siphonaxanthin/Carotenoid	Green algae *Codium cylindricum*	Diet induced obese mice	Decrease expression of lipogenesis-related genes and increase expression of energy expenditure-related genes in adipose tissue.	(31)

Uncoupling protein 1 (UCP1); Brown adipose tissue (BAT); Very low-density lipoproteins (VLDL); Peroxisome proliferator-activated receptor α (PPARα); Peroxisome proliferator-activated receptor γ (PPARγ); Sterol regulatory element-binding protein 1 (SREBP1).

Several studies have shown that the combination of marine bioactive compounds with anti-obesity activity has advantageous over individual compound via different additive or synergistic mechanisms (**Table 4**). There are several approaches to treat or prevent obesity including restricting glucose absorption, boosting body fat mobilization, increasing energy expenditure, reducing adipose tissue development, and blocking fat absorption (33). Therefore, one combinatorial mechanism is to target multiple pathways to enhance the response. In a study, Maeda and colleagues found that combining a bioactive compound with anti-obesity activities (fucoxanthin) and marine oil has an potential additive or beneficial anti-obesity effect. Fucoxanthin is a carotenoid found in edible brown seaweeds such as *Undaria pinnatifida* and *Hizikia fusiformis*. Fucoxanthin likely reduces the risk of obesity by lowering body fat in obese subjects (33). Fucoxanthin's mechanism of anti-obesity is currently ascribed to the up-regulation of mitochondrial uncoupling protein 1 (UCP1), which has a significant role in energy expenditure in white adipose tissue (34). Similar to fucoxanthin, fish oil can enhance energy expenditure by unregulated UCP1 expression in adipose tissue and significantly decrease weight gain and body fat (35). Fish oil also seems to stimulate lipid oxidation in healthy adults and limit triglyceride accumulation in adipose tissue through the regulation of fatty acid metabolism in the liver, and as a result, limit fat cell trophic growth (25, 26). The described activity from their combination is more effective for attenuating the weight gain of white adipose tissue than feeding with fucoxanthin alone in a diabetic/obese (KK-Ay) murine model. It has been suggested that the combination of fucoxanthin and fish oil can suppress the weight gain of white adipose tissue through multiple mechanisms. In other words, the individual compounds in the mixture might be able to attack different sites and/or different pathways associated with white adipose tissue maintenance (34). In another study, the authors further observed that the benefit of this combination could be due to another mechanism. For investigations depending on the oral administration of variables, the solubility of fucoxanthin is an essential factor to consider. The reason that fucoxanthin alone has lower anti-obesity activity compared to the mixture with fish oil could be due to the low solubility and oral bioavailability of the fucoxanthin. The authors suggested improving the oral bioavailability and absorption rate of fucoxanthin by first dissolving the carotenoid in fish oil or medium-chain triacylglycerols (MCT). As a result, Maeda et al. discerned that the anti-obesity activity of fucoxanthin with MCT was higher than fucoxanthin alone (36). The involvement of compounds that enhance the bioactive component solubility and stability is a significant type of synergism that is often undervalued (16).

In a similar study, lipids from *U. pinnatifida* (containing fucoxanthin) have anti-obesity activity both *in vivo* and *in vitro*, but due to low stability and bioavailability, Okada et al. (37) suggested developing a lipid delivery system in capsule form. The capsule was made of phospholipids derived from scallops (which also show anti-obesity activity). The results show that the combination of these bioactive lipids caused significant reductions in body weight and fat mass and has an additive effect compared to administering either lipid alone. The mechanism of reduction in the body weight might be due to the increases in the expression of UCP1 and UCP1 mRNA in epididymal fat tissue of diabetic/obese (KK-Ay) mice. The main reason behind the additive effect of the combination is that the scallop phospholipid increased the delivery and enhanced the stability and bioavailability of *U. pinnatifida* lipid in mice (38). Generally, in addition to pharmacodynamic synergy, several marine bioactive extracts may improve the solubility, absorption, distribution, or metabolism of active components with or without having particular pharmacological effects. The bioavailability of active components is increased as a result of these combined pharmacokinetic effects (16).

It is important to note that in the above-mentioned studies, the combined effects are not categorized as synergistic, however, but as “beneficial” or “additive” effects. Importantly, even the additive effect is not simply a summation of their constituents, and it requires the use of assessment models such as isobole or CI for calculating the interactions.

Mohamadi et al. (39) used the synergy assessment CI method to describe the type of combination effect occurring in their lipid accumulation study. Obesity is linked to an increased risk of NAFLD. The pathogenesis of NAFLD is due to several pathological events, such as insulin resistance, oxidative stress, apoptosis, and inflammation which enhance the fat accumulation in the liver. Therefore, using a combination of bioactive compounds targeting more than one pathological event may have higher efficacy for NAFLD therapy or prevention. The combination of polyunsaturated fatty acids (PUFAs) of fish oil and chicoric acid, consistent with this theory, reduced lipid accumulation in hepatic cells. Chicoric acid, a phenolic compound extracted from chicory leaves, has anti-oxidative and anti-inflammatory effects (40). PUFAs can suppress lipogenesis by targeting sterol regulatory element-binding protein-1 (SREBP-1), which is the main transcriptional regulator of lipid metabolism (41). As a result, the combination can have synergistic effects for NAFLD, associated with lipid accumulation and lipotoxicity. Based on Mohamedi et al. results, PUFAs and chicoric acid synergistically reduce lipid aggregation as observed by decreased oil red O staining and triglyceride levels in palmitate-induced hepatic HepG2 cells. The molecular mechanism of the synergy effect is the down-regulation of activated protein kinase (AMPK)-mediated (SREBP)-1/fatty acid synthase (FAS) and up-regulation of AMPK-mediated peroxisome proliferator-activated receptor α (PPARα)/uncoupling protein 2 (UCP2) signaling pathways (39).

Another additive or synergy mechanism could be considered to develop novel bioactive combinations is lowering the effective dose of agents to limit toxicity. Guo et al. (42) proved that the adverse effect of bioactive compounds, such as cytotoxicity and hemolytic activity, can be neutralized by using the desired bioactive compound in combination. Sea cucumber saponins (SCS) and eicosapentaenoic acid-enriched phospholipids (EPA-PL) are extracted from sea cucumber *Pearsonothuria graeffei* and *Cucumaria frondosa*, respectively. These two bioactive compounds could suppress lipogenesis, increase fatty acid oxidation, and lower lipid accumulation. However, the eicosapentaenoic acid is easily oxidized, and sea cucumber saponin has cytotoxic and hemolytic activities, which is recommended to use at a lower concentration. Guo et al. evaluated the synergistic impact of the combination at the ratio of 1:1 against orotic acid-induced NAFLD to minimize the side effects. According to their findings, the combination could significantly increase the expression of genes related to fatty acid β-oxidation, including peroxisome proliferator-activated receptor (PPARα), while significantly decreasing mRNA expression of genes involved in fatty acid biosynthesis, including fatty acid synthase (FAS), acetyl-CoA carboxylase (ACC), glucose-6-phosphate dehydrogenase (G6PDH), and malic enzyme. They made the conclusion that combining sea cucumber extracts had more protective benefits on NAFLD than using individual compounds alone (42). Although the authors described the interaction of the bioactive compounds as synergy, no proper synergistic assessment model has been used; therefore, the interpretation of this study may be questionable.

In conclusion, the solubility and stability of anti-obesity compounds of marine origin are the main drawbacks (43). Therefore, formulating combinations to address these concerns may be a useful strategy. Obesity is a complex, multifactorial disorder that can be treated or prevented by targeting multiple pathways; therefore, designing combinations that attack several molecular targets could be another advantageous strategy.

### Effects of bioactive compounds' interactions on anti-inflammatory and antioxidant activities

Inflammation is a physiological response to foreign organisms or tissue injuries. During the inflammatory response, several inflammatory cells, including macrophages, neutrophils, and lymphocytes, are activated. As a result, these cells release chemical mediators such as vasoactive amines, peptides, eicosanoids, acute-phase proteins, and cytokines (Tumor Necrosis Factor (TNF)-α, Interferon-gamma (IFNγ), nitric oxide (NO), prostaglandin E2 (PGE2) Interleukin (IL)-6 and IL-1β, IL-2, IL-8, IL-12, IL-17) to mediate the inflammatory response (44). In addition, oxidative stress can cause inflammation by producing reactive oxygen species (ROS). Excessive ROS production has been reported to trigger the inflammatory process resulting in the synthesis and secretion of pro-inflammatory cytokines through the activation of nuclear factor-kappa B/active protein-1 (NF-κB/AP-1) complex (45). If the inflammation continues for extended periods without resolution, chronic inflammation may eventually result in the development of other diseases, such as chronic asthma, rheumatoid arthritis, multiple sclerosis, inflammatory bowel disease, psoriasis, and cancer (3). Therefore, it is vital to treat and minimize long-term, unresolved inflammation with the use of anti-inflammatory and antioxidant agents. Although a few synthetic anti-inflammatory medicines exist, with continuous use, they all seem to have adverse physiological impacts. Lately, there has been an increasingly positive trend in the intake of antioxidants or anti-inflammatory products made from natural bioactive components (45). A large number of natural products have been extracted from marine organisms with anti-inflammatory and antioxidant properties, such as carotenoids, fucoidans, phlorotannins, sesquiterpenoids, diterpenes, steroids, polysaccharides, alkaloids, proteins, PUFAs, and other promising bioactive compounds for further research (Table 2) (5, 58).

**Table 2:** List of selected compounds isolated from marine sources with potential anti-inflammatory and anti-oxidant activity.

**Compound** **name/class**	**Marine source**	**Experimental method**	**Mechanism of action**	**References**
Lemnalol/Sesquiterpenoid	*Lemnalia cervicorni*	RAW264.7 macrophages & Carrageenan-activated rat paws	Inhibition of iNOS and COX-2 expression	(46)
Clovane compound 1/Sesquiterpenoid	*Rumphella antipathies*	FMLP/CB-activated human neutrophils	Inhibition of superoxide anions generation	(47)
8-hydroxybriaranes/Diterpenoids	Sea whip *Junceella fragilis*	RAW264.7 macrophages	Inhibition of COX-2 and iNOS release	(48)
Excavatolide B/Diterpene	*Briareum excavatum*	Carrageenan-activated rat paws	Inhibition of iNOS expression	(49)
Lobocrasol A/Diterpene	*Lobophytum crassum*	HepG2 cells	NF-κB activation	(50)
Solomonsterol A/Steroid (sterol)	*Theonella swinhoei*	Arthritis mice model	Reduction in arthritic score	(51)
Ergosta-7,22-dien-3-ol/Steroid (sterol)	*Marthasterias glacialis*	RAW264.7 macrophages	Inhibition of iNOS protein level	(52)
Steroid compound 5/Steroid (sterol)	*Astropecten polyacanthus*	Mice bone marrow-derived dendritic cells	IL-12 p40, IL-6, and TNF-α production	(53)
Sulfated polysaccharides/Polysaccharide	*Porphyra haitanensis*	Tropomyosin-induced mouse allergy model	Decrease in IgE level	(54)
Polysaccharides	*Digenea simplex*	Carrageenan-activated mouse paws	Reduction of edema volume	(52)
Indole-4-carboxaldehyde/Alkaloid	Brown seaweed *Sargassum thunbergii*	HepG2 cells	Limiting expression of pro-inflammatory genes	(55)
Lectin/Protein	*Caulerpa cupressoides*	Zymozan-activated rats	Reduction of leukocyte counts and myeloperoxidase activity	(56)
Hexadecanoic acid/Fatty acid 1-(15- methyl-1-oxohexadecyl)-pyrrolidine / Pyrrolidine	*Octopus vulgaris* ink extracts	RAW264.7 macrophages	Increase secretion of main cytokines from JAK-STAT, PI3K-Akt, and IL-17 pathways	(57)

Inducible nitric oxide synthase (iNOS); Cyclooxygenase-2 (COX-2); Nuclear factor-κB (NF-κB); Interleukin (IL)-12 IL-12 p40, IL-6, IL-17; Tumor necrosis factor α (TNFα); Immunoglobulin E (IgE); Janus kinase (JAK)-signal transducer and activator of transcription (STAT); Phosphoinositide-3-kinase–protein kinase B/Akt (PI3K-PKB/Akt).

For example, fish oil has well-recognized anti-inflammatory properties due to the presence of omega-3 PUFAs, especially docosahexaenoic acid (DHA) and eicosapentaenoic acid (EPA). However, one of the disadvantages is the sensitivity of n-3 PUFAs to oxidation (59). Lipid hydroperoxides are formed during digestion as a result of the oxidation of fatty acids. The formation of lipid oxidation products during digestion can be inhibited by using natural antioxidants in combination with a lipid co-constituent (60). Alternatively, carotenoids (such as astaxanthin) are lipid-soluble natural pigments in seaweed that have antioxidant activity due to their ability to quench singlet oxygen and scavenge free radicals. Accordingly, the adverse effects of oxidative sensitivity of bioactive compounds or extracts may be reduced by combining them with natural antioxidants, promoting additive or synergistic effects. Several *in vivo* and *in vitro* studies have been published to date that document the advantages of combining marine-derived antioxidant astaxanthin with fish oil and which conclude that this combination strategy is successful in enhancing the stability and efficacy of fish oil (61–63).

Otton et al. examined the effect of daily ingestion of fish oil/astaxanthin in combination and as individual compounds on oxidative stress parameters and the functional properties of lymphocytes isolated from Wistar rat's lymph nodes. The effects have been reported as reduced intracellular calcium concentration, reduced T- and B-lymphocyte proliferative capacity, enhanced enzymatic antioxidant capacity, and decreased superoxide anion, hydrogen peroxide, and NO• production. Therefore, the combination of astaxanthin and fish oil may have a slightly beneficial effect in preventing oxidative stress induced by PUFAs (62). This combination has also been investigated *in vivo* by Barros et al. (61) and leads to a similar conclusion based on examining the effect on oxidative stress and functional indexes of rat-isolated neutrophils. The mixture of fish oil/astaxanthin induced hypolipidemic/hypocholesterolemic effects in plasma and improved the phagocytic activity of activated neutrophils compared to the added astaxanthin or fish oil alone. The combination enhances the immune response by improving the glutathione-based redox balance in rat plasma and neutrophils. However, the study concluded that the effects of this combination were summative rather than synergistic. These studies have shown that the combined effect might not serve to improve the efficacy of a given active compound but rather act to reduce the adverse effects that the active agent may cause (16).

The use of isolated bioactive components or extracts at concentrations greater than their physiological ones is a concern in most *in vitro* investigations. However, *in vivo*, bioactive compounds are found in plasma or tissues at lower levels. Another advantage of using marine antioxidant compounds in combination is that mixtures can significantly decrease the dose of each antioxidant needed to achieve an effect (16). Natural antioxidants alone usually are effective at considerably higher concentrations. Using appropriate combinations of different natural/synthetic antioxidants, which can function synergistically, can solve this issue. The mixture of antioxidants might increase the antioxidant activity and reduce the dose of a single natural antioxidant in higher concentrations (16). Todorova et al. evaluated the interactions between the natural antioxidants taxifolin (a flavonoid) and fucoidan. The ABTS radical cation decolorization assay was used to determine the antioxidant capacity of pure taxifolin, fucoidan and their combinations. The study aimed to minimize potential adverse effects from the overuse of a single antioxidant and to evaluate the synergistic activity of combinations by the use of this chemical method (23) (The advantages and disadvantages of using chemical-based methods are discussed in more detail in the discussion section). In this decolorization study, only one chemical method has been used to assess the antioxidant effect. The antioxidant activity involves a complex process that is regulated by numerous mechanisms. When assessing the antioxidant activity of individual substances or in combination, more than one test is required due to the complexity of the measured antioxidant's capacities. This fact is exemplified by Saw et al. who, besides utilizing only the chemical method, also confirmed the antioxidant activity of the tested combination by measuring glutathione levels and a total antioxidant power *in vitro* cell assay (63).

Several studies have investigated the interactions between marine extracts in terms of synergistic or additive anti-inflammatory activity. For example, phlorotannins of *Ecklonia cava* and sulfated polysaccharides and crude extracts of *Sargassum horneri* have anti-inflammatory effects under *in vitro* and *in vivo* conditions (64–66). Sanjeewa et al. suggest that the combination of *E. cava* and *S. horneri* extracts have higher anti-inflammatory activity than *E. cava* or *S. horneri* extracts alone. The 8:2 combination of *E. cava : S. horneri* extracts significantly inhibited the inflammatory mediators (iNOS, COX-2, IL-1β, IL-6, and TNF-α) in lipopolysaccharides-activated RAW 264.7 macrophage cells compared to the single extracts (66). In other words, this interaction can boost the mixture's activity compared to individual compounds. They concluded that the synergistic effect of the combination could be due to the different bioactive properties of active compounds present in the extracts. The studies that examine the interaction between marine extracts provide more insight into the combined effect of several kinds of bioactive compounds. However, they cannot establish the synergy of individual compounds. It is suggested to identify the active compounds in the extracts, and single compounds are tested solely and in combination.

Correspondingly, marine extracts with anti-inflammatory or antioxidant activity could also be a source of marine-based prebiotics to combine with probiotics for developing more effective dietary supplements. Such symbiotic combinations may have a greater effect on improving intestinal health and avoiding inflammation of the intestines. *Gracilaria coronopifolia* seaweed extract, as a prebiotic, was combined with several probiotics (*Lactobacili* and *Bifidobacteria*), and the synergistic efficacy of the mixture was evaluated by Li et al. using the intestinal Caco-2 cell line model. Based on the result of the study, *the G. coronopifolia*/probiotic mixture inhibits ROS production under oxidative stress, reduces the damage of cells with oxidative stress, and inhibits the inflammatory response in cells. In addition, the combination can protect, maintain, and improve the function of intestinal cells by inhibiting the production of inflammatory factor substances (interleukin 8, IL-8) (67).

In general, inflammation and oxidative stress, which results in the production of chemical mediators and reactive oxygen species (free radicals), are linked to several chronic illnesses, including cancer, cardiovascular disease, and type 2 diabetes mellitus; therefore, designing combinations including bioactive compounds with both anti-inflammatory and antioxidant activities can be a promising scheme in chronic disease treatment and prevention.

### Effects of bioactive compounds' interactions on anticarcinogenic activity

Cancer development increases with age, and the beginning of cancer is often a slow process. Consuming functional food with anti-cancer properties is considered a chemo-preventative approach to offset or reduce the chance of cancer development (68). Chemo-preventative nutraceuticals inhibit the development of a healthy cell into a cancer cell by inducing antioxidant and anti-inflammatory activity, inactivation of phase I enzymes, and induction of phase II enzymes. Chemotherapeutic nutraceuticals, on the other hand, target cancer cells directly by inhibiting tumor growth (inhibiting cell proliferation, inducing cell differentiation, and apoptosis) or inhibiting tumor spread (inhibiting tumor invasion, anti-metastasis) (6).

Bioactive compounds such as polyphenols, polysaccharides, alkaloids, peptides, and terpenoids have been isolated from marine organisms and elicit anti-cancer activity *in vitro* and *in vivo* (Table 3). The main source of these isolated bioactive compounds is marine microbiota, including microalgae, fungi, seaweeds, mangroves, bacteria, cyanobacteria, and actinobacteria. The mechanism of action is mainly through inhibiting tumor growth *via* augmenting and supporting apoptosis, necrosis, and lysis of the tumor cells (82).

**Table 3:** List of selected marine bioactive compounds with anti-cancer activity.

**Compound** **name/class**	**Marine source**	**Experimental method**	**Mechanism of action**	**References**
Fucoidan/Polysaccharides Laminarin/Polysaccharides	*Sargassum hemiphyllum*	Lung tissue from mice	Altered the expression patterns of inflammatory cytokines, reducing radiation pneumonitis and radiotherapy-induced lung fibrosis	(69)
	*Laminaria digitata*	Human colon cancer cells (HT-29)	Induces apoptosis & suppresses ErbB signaling pathway activation	(70)
Dieckol/Polyphenol	*Ecklonia cava*	HT1080 cells	Downregulates FAK signaling pathway mediated by ROS	(71)
Diphlorethohydroxycarmalol/ Phlorotannin	*Eisenia bicyclis*	HL60 cells	Induces apoptosis & reduces Bcl-2 expression & depletes mitochondrial membrane potential	(72)
HFGP/Glycoprotein	*Hizikia fusiformis*	HepG2 cells	Induces apoptosis & upregulates expressions of Fas, Fas-associated death domain protein, Bax, and Bad	(73)
		SMMC-7721 cells	Induces apoptosis *via* ROS-mediated mitochondrial pathway	(74)
Fucoxanthin/Carotenoid	Brown algae	Carcinogenic murine azoxymethane/dextran sodium sulfate (AOM/DSS) mice model	Exhibit chemopreventive potential by downregulation of HSP70 genes	(75)
Pheophorbide a/Chlorophyll	*Grateloupia elliptica*	Glioblastoma cells (U87 MG)	Induces cytostatic activity	(76)
Clerosterol/Sterol	*Codium fragile*		Induces apoptosis & increase of Bax & decrease of Bcl-2 expression & activates Caspase-3/Caspase-9	(77)
Astropectenols/Sterol	*Astropecten polyacanthus*	HL-60 cell line	Induces caspase dependent apoptosis & regulates Bcl-2, Bax, PARP	(53)
Pleurocidin-Amide/Peptides	*Pleuronectes americanus*	A549 lung adenocarcinoma cells	Inhibit autophagy of A549 cells, and induce apoptosis	(78)
Aplysin/Terpenoids	*Aplysia kurodai*	A549 NSCLC cell line	Anti-neoplastic agent & induces intrinsic/extrinsic apoptosis	(79)
Mandelalide A-D/Polyketides	*Lissoclinum mandelai*	Proliferating tumor cells	Induces intrinsic apoptosis & inhibits mammalian ATP synthase complex V	(80)
N-(2-ozoazepan-3-yl)-pyrrolidine-2-carboxamide/Pyrrolidine	*Octopus vulgaris* ink extracts	Human cancer cell lines (22Rv1, HeLa, A549)	Anti-proliferative, early-apoptosis induction, reactive species modulation, and nuclei disruption in 22Rv1 cells	(81)

Focal adhesion kinase (FAK); Reactive oxygen species (ROS); Heat shock protein 70 (HSP70).

Marine bioactive compounds or extracts with antioxidant activity could be a decent candidate to prevent cancer development since many chronic diseases, including cancer, are caused by oxidative stress (63). Astaxanthin has antioxidant activity and inhibits oxidative damage, which could explain its chemo-preventative activity. Accordingly, Saw et al. suggested that a lower concentration of astaxanthin has potent antioxidant activity in conjunction with PUFAs (DHA or EPA) and demonstrates synergistic antioxidant effects. Inducing the nuclear factor erythroid 2-related factor 2/ antioxidant response elements (Nrf2/ARE) pathway in an *in vitro* HepG2-C8-ARE-luciferase cell line, beneficial synergism is aligned with the pathway which has the main role in the induction of antioxidant genes that protect against oxidative damage (63). As a result, the authors have suggested that this combination is a promising cancer chemoprevention strategy. In this study, the synergism has been confirmed by using CI method.

The mechanism of synergy in Saw et al. (63) study is to reduce the concentration of bioactive compounds in combination to produce better efficacy with less toxicity than individual compounds. However, cancer is a multi-targetable disease, and the combination of compounds that address many targets simultaneously can be a better approach to treat various types of cancer. Designing marine-based combinations that promote cell death, inhibit cell proliferation and invasion, and boost the immune system simultaneously are future possibilities. Moreover, high biological activity and the wide availability of these marine compounds are advantages that should be considered (83). Most studies focus on the synergistic effect of marine compounds in combination with existing anti-cancer drugs and enhancing drug therapy (84–86). Investigating the synergistic interaction between naturally occurring bioactive compounds or extracts with anti-cancer activity is highly recommended.

## Discussion and future perspective

Several combinations of marine compounds and extracts have been studied, and their interaction mechanism has been discussed in this review. Of all mechanisms, enhancing the bioavailability and reducing adverse effects of active compounds are the most frequently reported. However, in the case of chronic non-communicable diseases that involve different pathogenesis pathways, the ideal combination may be applying compounds with non-overlapping activities to provide either additive or synergistic response on multiple molecular targets. Inflammation and oxidative stress are the two main aspects of chronic diseases. Therefore, combinations that target pathways in relation to these two aspects are considered useful approaches for therapy or prevention. Future research should concentrate on finding such combinations.

The diversity of the marine compounds or extracts examined in combinations should be increased. Most studies have focused on fish oil and carotenoids (mostly fucoidan, astaxanthin, and fucoxanthin) to develop novel combinations (Table 4). Future research should investigate the combinatorial effect of other promising marine bioactive compounds. In recent years, attention has been drawn to a greater extent to marine proteins and peptides. These bioactive compounds have a range of biological properties, including immunomodulating, neuroprotective, anti-diabetic, anti-cancer, antiviral, and anti-oxidative aspects (88, 89). Despite their significant bioactivity, there are several problems to use these compounds in functional foods and nutraceuticals, such as poor solubility, unfavorable taste, low bioavailability, and stability (1). Applying peptide or protein in combination with other bioactive compounds or extracts may be a solution to improving their water solubility, increasing their bioavailability, improving sensory properties, increasing their food matrix compatibility, improving their stability in the gastrointestinal tract, and protecting bioactive components from unfavorable food matrix environment.

**Table 4:** List of combination of marine bioactive compounds.

**Compound name**	**Experimental method**	**Mechanism of action**	**Combination effect**	**Combination effects assessment**	**References**
**Anti-obesity activity**					
Fucoxanthin & fish oil	Diabetes/obesity mouse model (KK-Ay).	Plural mechanisms	Beneficial effect on the attenuation of WAT weight gain	Potential additive or beneficial	(34)
Fucoxanthin & MCT	Diabetes/obesity mouse model (KK-Ay).	Up-regulation of mitochondrial UCP1	Enhanced anti-obesity effects	Enhancement or beneficial	(36)
			Increasing the bioavailability of fucoxanthin		
Fucoxanthin & phospholipids of scallop	KK-Ay mice	Increases in the expression of UCP1 and UCP1 mRNA	Significant reductions in body weight and fat mass	Potential additive effect	(38)
Fish oils & chicoric acid	Hepatic HepG2 cells	Down-regulation of AMPK-mediated SREBP-1/FAS and up-regulation of AMPK-mediated PPARα/UCP2 signaling pathways	Synergistically reduce lipid aggregation	Synergistic (CI)	(39)
Sea cucumber saponins & eicosapentaenoic acid-enriched phospholipids	Orotic acid-induced NAFLD rats	Increase expression of PPARα. Decreasing mRNA expression of FAS, ACC, G6PDH, malic enzyme	Synergistic effect on lipogenesis inhibition and β-oxidation enhancement	Potential synergistic (No synergy analysis)	(42)
**Anti-inflammatory and Antioxidant activity**					
Fish oil (PUFAs) & astaxanthin	Wistar rats Lymphocytes	Reduced proliferative capacity of T- and B-lymphocytes, Minimize superoxide anion, hydrogen peroxide, and NO• production	Preventing Lipid hydroperoxide	Enhancement or beneficial	(62)
			Preventing oxidative stress caused by PUFAs		
			Stimulating the immuno-modulatory effects of fish oil		
Fish oil (PUFAs) & astaxanthin	Wistar rats neutrophils	Improving the glutathione-based redox balance	Induced hypolipidemic/hypocholesterolemic effects in plasma	Enhancement or beneficial	(61)
			Improved the phagocytic activity of activated neutrophils		
Astaxanthin & omega-3 fatty acids	HepG2-C8-A cell line	Inducing the Nrf2/ARE pathway	Synergistic antioxidant Protect against oxidative stress	Synergistic (CI)	(63)
Taxifolin (flavonoid) & fucoidan	Chemical method (ABTS)		Decrease the dose needed of each bioactive compound	Synergistic	(23)
				(Isobole method)	
*Gracilaria coronopifolia* seaweed extract & probiotic	Caco-2 cell line		Improve intestinal health	Enhancement or beneficial	(67)
			Avoid inflammation of the intestines		
Phlorotannins of *E. cava* & sulfated polysaccharides and crude extracts of *S. horneri*	RAW 264.7 macrophage cells	Blocking NF-κB and MAPK pathways	Synergistic anti-inflammatory effects	Potential synergistic (No synergy analysis)	(87)
**Chemopreventive activity**					
Astaxanthin & omega-3 fatty acids	HepG2-C8-A cell line	Inducing the Nrf2/ARE pathway	Synergistic antioxidant Protect against oxidative stress	Synergistic (CI)	(63)

NF-E2-related factor 2 (Nrf2); Antioxidant response element (ARE); Mitogen-activated protein kinases (MAPKs); Focal adhesion kinase (FAK); Acetyl CoA carboxylase (ACC); Glucose-6-phosphate dehydrogenase (G6PD).

Moreover, less attention has been paid to design combinations with anti-cancer activity. To our knowledge, unfortunately, no study has yet been published which investigates the synergistic effect of naturally occurring marine bioactive or marine extracts as chemotherapeutic or chemopreventive functional foods. This research gap should be filled by encouraging more studies to develop novel combinations with anti-cancer activities. The current repository of studies on the combination of phytochemicals with defined anti-cancer activity (which show promising results both *in vitro* and *in vivo*) may serve as guides to future investigations (12–15).

In addition, the combinatorial effects of marine bioactive components in the human body are currently lacking as a source of information. Most marine combination studies have been carried out as *in vitro* (cell culture assays, for example) or *in vivo* (animal) models; therefore offer limited information regarding the bioavailability and biotransformation of the components in the human body after consumption.

Synergistic, additive, or antagonistic factors of the interactions in combinations must be determined to avoid misconceptions in developed synergy. Some studies do not categorize the combinational interaction or distinguish between synergistic and additive effects. The synergistic and additive effects are two different interactions that should not be confused. Advantageously, the combined effect should be clearly and accurately defined. Consequently, to identify the combined effect and to facilitate the comparison of results between relevant research, it is recommended to use analytical methods such as Isobole analysis or CI (16).

Developing novel natural bioactive combinations is likely a complicated and time-consuming project. Several factors should be considered before planning the investigation. The first factor to consider is the knowledge of the mechanism of action of selected individual compounds and their combinations. Since the mode of action of a combination could be different from that of the same compounds acting individually, understanding the mechanism of synergistic effects of combinations is important for the future development of new marine bioactive combinations and functional food (16). For example, sulphated polysaccharides extracted from the seaweeds *Ulva clathrate* and *Cladosiphon okamuranus* (fucoidan) have antiviral properties against paramyxovirus infection. However, the combination has no clear synergistic advantage, and the *U. clathrate* extract antagonizes the effect of fucoidan on the viral attachment/entry in the mixture, probably because both act on the same target (90).

Selecting an appropriate model for testing the combined effect is the main step to obtaining relevant data for understanding the combination effects. The cell-free chemical model is not a convenient method to test the combinational effect. It mainly relies on the solvent (in which the compounds are dissolved for the experiment), the concentration and volume ratio of the individual compounds, and the estimation method for testing the combination effect. As a result, the data concluded from the chemical method may not be relevant to biological systems (e.g., cell culture, animals, and humans). Consequently, this may lead to incorrect conclusions and mislead the study (91). Improving overall conditions for the experimental context, *in vivo* animal models replicate the physiology of an entire organism. However, this type of model has several drawbacks. Inconsistency in model responses due to species variations, difficulties in interpreting results to humans, dealing with ethical issues, and the effort required to maintain an animal facility are prominent issues. Therefore, the advanced cell culture method, such as co-culture, can be an alternative method to animal testing. In this model, cell culture is conducted by using one or more cell lines in (co-) culture under two-dimensional (2D) settings. Co-culture can more closely resemble an *in vivo* model, allowing for the efficient and reproducible investigation of molecular mechanisms of combinations and uncovering multi-target mechanisms (92). For instance, co-culture models of immune and intestinal cells can be developed to study the anti-inflammatory/antioxidant activities of the food combination in relation to the compounds' absorbance and bioavailability after digestion in a cell model (93, 94). Due to technological advancements, three-dimensional (3D) cell culture has also been developed to imitate the complex composition of human tissue and organs. This model can be used for in-depth investigations of molecular mechanisms of combinational effects (95).

## Conclusion

Marine functional foods and combination therapy have increasingly been recognized and studied as viable and successful approaches for treating or preventing chronic non-communicable diseases. Several combinations of marine-based bioactive compounds or extracts have been proven to possess synergistic or enhanced therapeutic effects, notably antioxidant, anti-inflammatory, anti-obesity, and chemo-preventative activities. The most often stated benefits of combinations involve increasing the bioavailability of compounds and minimizing their adverse side effects. The best combinatorial approach, however, should focus on targeting multiple pathways of chronic non-communicable diseases. In addition, there are many marine bioactive compounds with known biological properties, but only a few have been tested in combination. The limited research in the field of marine natural combinations provides an incredible opportunity for researchers to develop novel functional foods exhibiting higher efficacy within the context of disease prevention and treatment. However, for developing novel marine bioactive combinations, several factors should be considered to ensure confidence in the applicability of the tested combinations.

## Author contributions

BE performed the literature search and wrote the original manuscript. SB edited the original manuscript. JLi, TY, and BZ edited the final version of the manuscript. JLu conceived the idea and edited the final version of the manuscript. All authors contributed to the article and approved the submitted version.

## References

[1] Hosseini SF Ramezanzade L McClements DJ. Recent advances in nanoencapsulation of hydrophobic marine bioactives: bioavailability, safety, and sensory attributes of nano-fortified functional foods. Trends Food Sci Technol. (2021) 109:322–39. 10.1016/j.tifs.2021.01.045

[2] Martirosyan D Singh J. A new definition of functional food by FFC: what makes a new definition unique? Funct Foods Health Dis. (2015) 5:209–23. 10.31989/ffhd.v5i6.183

[3] Suleria HAR Gobe G Masci P Osborne SA. Marine bioactive compounds and health promoting perspectives) innovation pathways for drug discovery. Trends Food Sci Technol. (2016) 50:44–55. 10.1016/j.tifs.2016.01.019

[4] Barreca M Spano V Montalbano A Cueto M Diaz Marrero AR Deniz I. Marine anticancer agents: an overview with a particular focus on their chemical classes. Mar Drugs. (2020) 18:0619. 10.3390/md18120619 33291602 PMC7761941

[5] Cheung RCF Ng TB Wong JH Chen Y Chan WY. Marine natural products with anti-inflammatory activity. Appl Microbiol Biotechnol. (2016) 100:1645–66. 10.1007/s00253-015-7244-3 26711278

[6] Santana-Gálvez J Cisneros-Zevallos L Jacobo-Velázquez DA. A practical guide for designing effective nutraceutical combinations in the form of foods, beverages, and dietary supplements against chronic degenerative diseases. Trends Food Sci Technol. (2019) 88:179–93. 10.1016/j.tifs.2019.03.026

[7] Ronis MJJ Pedersen KB Watt J. Adverse effects of nutraceuticals and dietary supplements. Annu Rev Pharmacol Toxicol. (2018) 58:583–601. 10.1146/annurev-pharmtox-010617-052844 28992429 PMC6380172

[8] Braithwaite MC Tyagi C Tomar LK Kumar P Choonara YE Pillay V. Nutraceutical-based therapeutics and formulation strategies augmenting their efficiency to complement modern medicine: an overview. J Funct Foods. (2014) 6:82–99. 10.1016/j.jff.2013.09.022

[9] Marinangeli CP Jones PJ. Functional food ingredients as adjunctive therapies to pharmacotherapy for treating disorders of metabolic syndrome. Ann Med. (2010) 42:317–33. 10.3109/07853890.2010.484026 20486826

[10] Gertsch J. Botanical drugs, synergy, and network pharmacology: forth and back to intelligent mixtures. Planta Med. (2011) 77:1086–98. 10.1055/s-0030-1270904 21412698

[11] Huang X Kong L Li X Chen X Guo M Zou H. Strategy for analysis and screening of bioactive compounds in traditional Chinese medicines. J Chromatogr B Analyt Technol Biomed Life Sci. (2004) 812:71–84. 10.1016/S1570-0232(04)00546-X 15556489

[12] Singh CK Siddiqui IA El-Abd S Mukhtar H Ahmad N. Combination chemoprevention with grape antioxidants. Mol Nutr Food Res. (2016) 60:1406–15. 10.1002/mnfr.201500945 26829056 PMC4911802

[13] Malongane F McGaw LJ Mudau FN. The synergistic potential of various teas, herbs and therapeutic drugs in health improvement: a review. J Sci Food Agric. (2017) 97:4679–89. 10.1002/jsfa.8472 28585285

[14] Phan MAT Paterson J Bucknall M Arcot J. Interactions between phytochemicals from fruits and vegetables: Effects on bioactivities and bioavailability. Crit Rev Food Sci Nutr. (2018) 58:1310–29. 10.1080/10408398.2016.1254595 27880063

[15] van Breda SGJ de Kok T. Smart combinations of bioactive compounds in fruits and vegetables may guide new strategies for personalized prevention of chronic diseases. Mol Nutr Food Res. (2018) 62:597. 10.1002/mnfr.201700597 29108107

[16] Caesar LK Cech NB. Synergy and antagonism in natural product extracts: when 1 + 1 does not equal 2. Nat Prod Rep. (2019) 36:869–88. 10.1039/C9NP00011A 31187844 PMC6820002

[17] Chen X Li H Zhang B Deng Z. The synergistic and antagonistic antioxidant interactions of dietary phytochemical combinations. Crit Rev Food Sci Nutr. (2022) 62:5658–77. 10.1080/10408398.2021.1888693 33612011

[18] Chou TC. Theoretical basis, experimental design, and computerized simulation of synergism and antagonism in drug combination studies. Pharmacol Rev. (2006) 58:621–81. 10.1124/pr.58.3.10 16968952

[19] Tang J Wennerberg K Aittokallio T. What is synergy? The Saariselka agreement revisited. Front Pharmacol. (2015) 6:181. 10.3389/fphar.2015.00181 26388771 PMC4555011

[20] Zhao L Au JL Wientjes MG. Comparison of methods for evaluating drug-drug interaction. Front Biosci (Elite Ed). (2010) 2:241–9. 10.2741/e86 20036874 PMC2885905

[21] Leena MM Silvia MG Vinitha K Moses JA Anandharamakrishnan C. Synergistic potential of nutraceuticals: mechanisms and prospects for futuristic medicine. Food Funct. (2020) 11:9317–37. 10.1039/D0FO02041A 33211054

[22] Moran-Santibanez K Cruz-Suarez LE Ricque-Marie D Robledo D Freile-Pelegrin Y Pena-Hernandez MA. Synergistic effects of sulfated polysaccharides from mexican seaweeds against measles virus. Biomed Res Int. (2016) 2016:8502123. 10.1155/2016/8502123 27419139 PMC4933867

[23] Todorova MN Kiselova-Kaneva YD Potoroko IY Kalinina IV Ivanova DG Galunska BT. Antioxidant activity of taxifolin derived from larch: synergistic studies. Bulgarian Chem Commun. (2019) 51:172–6.

[24] Baillie RA Takada R Nakamura M Clarke SD. Coordinate induction of peroxisomal acyl-CoA oxidase and UCP-3 by dietary fish oil: a mechanism for decreased body fat deposition. Prostaglandins Leukot Essent Fatty Acids. (1999) 60:351–6. 10.1016/S0952-3278(99)80011-8 10471120

[25] Couet C Delarue J Ritz P Antoine JM Lamisse F. Effect of dietary fish oil on body fat mass and basal fat oxidation in healthy adults. Int J Obes Relat Metab Disord. (1997) 21:637–43. 10.1038/sj.ijo.0800451 15481762

[26] Parrish CC Pathy DA Angel A. Dietary fish oils limit adipose tissue hypertrophy in rats. Metabolism. (1990) 39:217–9. 10.1016/0026-0495(90)90038-E 2308514

[27] Nakatani T Kim HJ Kaburagi Y Yasuda K Ezaki O A. low fish oil inhibits SREBP-1 proteolytic cascade, while a high-fish-oil feeding decreases SREBP-1 mRNA in mice liver: relationship to anti-obesity. J Lipid Res. (2003) 44:369–79. 10.1194/jlr.M200289-JLR200 12576519

[28] Lee HG Jayawardena TU Song KM Choi YS Jeon YJ Kang MC. Dietary fucoidan from a brown marine algae (Ecklonia cava) attenuates lipid accumulation in differentiated 3T3-L1 cells and alleviates high-fat diet-induced obesity in mice. Food Chem Toxicol. (2022) 162:112862. 10.1016/j.fct.2022.112862 35157925

[29] Xu H Wang J Zhang X Li Z Wang Y Xue C. Inhibitory effect of fucosylated chondroitin sulfate from the sea cucumber Acaudina molpadioides on adipogenesis is dependent on Wnt/beta-catenin pathway. J Biosci Bioeng. (2015) 119:85–91. 10.1016/j.jbiosc.2014.05.026 24982018

[30] Kimura M Iida M Yamauchi H Suzuki M Shibasaki T Saito Y. Astaxanthin supplementation effects on adipocyte size and lipid profile in OLETF rats with hyperphagia and visceral fat accumulation. J Funct Foods. (2014) 11:114–20. 10.1016/j.jff.2014.08.001

[31] Li ZS Zheng JW Manabe Y Hirata T Sugawara T. Anti-obesity properties of the dietary green alga, codium cylindricum, in high-fat diet-induced obese mice. J Nutr Sci Vitaminol. (2018) 64:347–56. 10.3177/jnsv.64.347 30381625

[32] Trigueros L Pena S Ugidos AV Sayas-Barbera E Perez-Alvarez JA Sendra E. Food ingredients as anti-obesity agents: a review. Crit Rev Food Sci Nutr. (2013) 53:929–42. 10.1080/10408398.2011.574215 23768185

[33] Hu X Tao N Wang X Xiao J Wang M. Marine-derived bioactive compounds with anti-obesity effect: a review. J Funct Foods. (2016) 21:372–87. 10.1016/j.jff.2015.12.00628571534

[34] Maeda H Hosokawa M Sashima T Miyashita K. Dietary combination of fucoxanthin and fish oil attenuates the weight gain of white adipose tissue and decreases blood glucose in obese/diabetic KK-Ay mice. J Agric Food Chem. (2007) 55:7701–6. 10.1021/jf071569n 17715888

[35] Kawada T Kayahashi S Hida Y Koga K-j Nadachi Y Fushiki T. Fish (Bonito) oil supplementation enhances the expression of uncoupling protein in brown adipose tissue of rat. J Agricult Food Chem. (1998) 46:1225–7. 10.1021/jf9711000

[36] Maeda H Hosokawa M Sashima T Funayama K Miyashita K. Effect of medium-chain triacylglycerols on anti-obesity effect of fucoxanthin. J Oleo Sci. (2007) 56:615–21. 10.5650/jos.56.615 17992001

[37] Ruzickova J Rossmeisl M Prazak T Flachs P Sponarova J Veck M. Omega-3 PUFA of marine origin limit diet-induced obesity in mice by reducing cellularity of adipose tissue. Lipids. (2004) 39:1177–85. 10.1007/s11745-004-1345-9 15736913

[38] Okada T Mizuno Y Sibayama S Hosokawa M Miyashita K. Antiobesity effects of Undaria lipid capsules prepared with scallop phospholipids. J Food Sci. (2011) 76:H2–6. 10.1111/j.1750-3841.2010.01878.x 21535684

[39] Mohammadi M Abbasalipourkabir R Ziamajidi N. Fish oil and chicoric acid combination protects better against palmitate-induced lipid accumulation via regulating AMPK-mediated SREBP-1/FAS and PPARalpha/UCP2 pathways. Arch Physiol Biochem. (2020) 5:1–9. 10.1080/13813455.2020.1789881 32654534

[40] Lee J Scagel CF. Chicoric acid: chemistry, distribution, and production. Front Chem. (2013) 1:40. 10.3389/fchem.2013.00040 24790967 PMC3982519

[41] Tai CC Ding ST. N-3 polyunsaturated fatty acids regulate lipid metabolism through several inflammation mediators: mechanisms and implications for obesity prevention. J Nutr Biochem. (2010) 21:357–63. 10.1016/j.jnutbio.2009.09.010 20149625

[42] Guo Y Han X Che H Li Z Dong P Xue C. Synergistic effect of eicosapentaenoic acid-enriched phospholipids and sea cucumber saponin on orotic acid-induced non-alcoholic fatty liver disease in rats. Royal Soc Open Sci. (2018) 5:172182. 10.1098/rsos.172182 30109054 PMC6083717

[43] Wan-Loy C Siew-Moi P. Marine algae as a potential source for anti-obesity agents. Mar Drugs. (2016) 14:222. 10.3390/md14120222 27941599 PMC5192459

[44] Abdulkhaleq LA Assi MA Abdullah R Zamri-Saad M Taufiq-Yap YH Hezmee MNM. The crucial roles of inflammatory mediators in inflammation: a review. Vet World. (2018) 11:627–35. 10.14202/vetworld.2018.627-635 29915501 PMC5993766

[45] Lu CC Yen C. Antioxidative and anti-inflammatory activity of functional foods. Curr Opin Food Sci. (2015) 2:1–8. 10.1016/j.cofs.2014.11.002

[46] Lee HP Huang SY Lin YY Wang HM Jean YH Wu SF. Soft coral-derived lemnalol alleviates monosodium urate-induced gouty arthritis in rats by inhibiting leukocyte infiltration and iNOS, COX-2 and c-Fos protein expression. Mar Drugs. (2013) 11:99–113. 10.3390/md11010099 23306170 PMC3564160

[47] Chung HM Wang WH Hwang TL Wu YC Sung PJ. Natural clovanes from the gorgonian coral Rumphella antipathies. Nat Prod Commun. (2013) 8:1037–40. 10.1177/1934578X1300800801 24079161

[48] Chen YY Fang LS Chen YH Peng BR Su TP Huynh TH. New 8-hydroxybriaranes from the gorgonian coral junceella fragilis (Ellisellidae). Mar Drugs. (2019) 17:534. 10.3390/md17090534 31540107 PMC6780648

[49] Lin YY Lin SC Feng CW Chen PC Su YD Li CM. Anti-inflammatory and analgesic effects of the marine-derived compound excavatolide B isolated from the culture-type formosan gorgonian briareum excavatum. Mar Drugs. (2015) 13:2559–79. 10.3390/md13052559 25923315 PMC4446594

[50] Thao NP Luyen BT Ngan NT Song SB Cuong NX Nam NH. New anti-inflammatory cembranoid diterpenoids from the Vietnamese soft coral Lobophytum crassum. Bioorg Med Chem Lett. (2014) 24:228–32. 10.1016/j.bmcl.2013.11.033 24314396

[51] Mencarelli A D'Amore C Renga B Cipriani S Carino A Sepe V. Solomonsterol A, a marine pregnane-X-receptor agonist, attenuates inflammation and immune dysfunction in a mouse model of arthritis. Mar Drugs. (2013) 12:36–53. 10.3390/md12010036 24368568 PMC3917259

[52] Pereira DM. Correia-da-Silva G, Valentao P, Teixeira N, Andrade PB. Anti-inflammatory effect of unsaturated fatty acids and Ergosta-7,22-dien-3-ol from Marthasterias glacialis: prevention of CHOP-mediated ER-stress and NF-kappaB activation. PLoS ONE. (2014) 9:e88341. 10.1371/journal.pone.0088341 24551093 PMC3923769

[53] Thao NP Cuong NX Luyen BT Nam NH Cuong PV Thanh NV. Steroidal constituents from the starfish Astropecten polyacanthus and their anticancer effects. Chem Pharm Bull. (2013) 61:1044–51. 10.1248/cpb.c13-00490 24088696

[54] Shi C Pan T Cao M Liu Q Zhang L Liu G. Suppression of Th2 immune responses by the sulfated polysaccharide from Porphyra haitanensis in tropomyosin-sensitized mice. Int Immunopharmacol. (2015) 24:211–8. 10.1016/j.intimp.2014.11.019 25499728

[55] Cha SH Hwang Y Heo SJ Jun HS. Indole-4-carboxaldehyde isolated from seaweed, sargassum thunbergii, attenuates methylglyoxal-induced hepatic inflammation. Mar Drugs. (2019) 17:486. 10.3390/md17090486 31438528 PMC6780312

[56] da Conceicao Rivanor RL Chaves HV. do Val DR de Freitas AR Lemos JC Rodrigues JA. A lectin from the green seaweed Caulerpa cupressoides reduces mechanical hyper-nociception and inflammation in the rat temporomandibular joint during zymosan-induced arthritis. Int Immunopharmacol. (2014) 21:34–43. 10.1016/j.intimp.2014.04.009 24768528

[57] Hernández-Zazueta MS Luzardo-Ocampo I García-Romo JS Noguera-Artiaga L Carbonell-Barrachina Á A Taboada-Antelo P. Bioactive compounds from Octopus vulgaris ink extracts exerted anti-proliferative and anti-inflammatory effects in vitro. Food Chem Toxicol. (2021) 151:112119. 10.1016/j.fct.2021.112119 33722603

[58] Penalver R Lorenzo JM Ros G Amarowicz R Pateiro M Nieto G. Seaweeds as a functional ingredient for a healthy diet. Mar Drugs. (2020) 18:301. 10.3390/md18060301 32517092 PMC7345263

[59] Deckelbaum RJ Calder PC. Dietary n-3 and n-6 fatty acids: are there 'bad' polyunsaturated fatty acids? Curr Opin Clin Nutr Metab Care. (2010) 13:123–4. 10.1097/MCO.0b013e328336696d 20125001

[60] Maestre R Douglass JD Kodukula S Medina I Storch J. Alterations in the intestinal assimilation of oxidized PUFAs are ameliorated by a polyphenol-rich grape seed extract in an in vitro model and Caco-2 cells. J Nutr. (2013) 143:295–301. 10.3945/jn.112.160101 23325921 PMC3713019

[61] Barros MP Marin DP Bolin AP de Cassia Santos Macedo R Campoio TR Fineto C Jr. Combined astaxanthin and fish oil supplementation improves glutathione-based redox balance in rat plasma and neutrophils. Chem Biol Interact. (2012) 197:58–67. 10.1016/j.cbi.2012.03.005 22465178

[62] Otton R Marin DP Bolin AP de Cassia Santos Macedo R Campoio TR Fineto C Jr. Combined fish oil and astaxanthin supplementation modulates rat lymphocyte function. Eur J Nutr. (2012) 51:707–18. 10.1007/s00394-011-0250-z 21972007

[63] Saw CL Yang AY Guo Y Kong AN. Astaxanthin and omega-3 fatty acids individually and in combination protect against oxidative stress via the Nrf2-ARE pathway. Food Chem Toxicol. (2013) 62:869–75. 10.1016/j.fct.2013.10.023 24157545

[64] Kim ME Jung YC Jung I Lee HW Youn HY Lee JS. Anti-inflammatory effects of ethanolic extract from Sargassum horneri (Turner) C. Agardh on lipopolysaccharide-stimulated macrophage activation via NF-kappaB pathway regulation. Immunol Invest. (2015) 44:137–46. 10.3109/08820139.2014.942459 25140761

[65] Lee JH Ko JY Oh JY Kim CY Lee HJ Kim J. Preparative isolation and purification of phlorotannins from Ecklonia cava using centrifugal partition chromatography by one-step. Food Chem. (2014) 158:433–7. 10.1016/j.foodchem.2014.02.112 24731366

[66] Sanjeewa KKA Fernando IPS Kim SY Kim WS Ahn G Jee Y. Ecklonia cava (Laminariales) and Sargassum horneri (Fucales) synergistically inhibit the lipopolysaccharide-induced inflammation via blocking NF-κB and MAPK pathways. Algae. (2019) 34:45–56. 10.4490/algae.2019.34.2.10

[67] Li PH Lu WC Chan YJ Zhao YP Nie XB Jiang CX. Feasibility of using seaweed (*Gracilaria coronopifolia*) synbiotic as a bioactive material for intestinal health. Foods. (2019) 8:623. 10.3390/foods8120623 31783694 PMC6963959

[68] Saldanha SN Tollefsbol TO. The role of nutraceuticals in chemoprevention and chemotherapy and their clinical outcomes. J Oncol. (2012) 2012:192464. 10.1155/2012/192464 22187555 PMC3236518

[69] Yu HH Chengchuan Ko E Chang CL Yuan KS Wu ATH Shan YS. Fucoidan inhibits radiation-induced pneumonitis and lung fibrosis by reducing inflammatory cytokine expression in lung tissues. Mar Drugs. (2018) 16:392. 10.3390/md16100392 30347679 PMC6213111

[70] Park HK Kim IH Kim J Nam TJ. Induction of apoptosis and the regulation of ErbB signaling by laminarin in HT-29 human colon cancer cells. Int J Mol Med. (2013) 32:291–5. 10.3892/ijmm.2013.1409 23739740 PMC3776715

[71] Park SJ Jeon YJ. Dieckol from Ecklonia cava suppresses the migration and invasion of HT1080 cells by inhibiting the focal adhesion kinase pathway downstream of Rac1-ROS signaling. Mol Cells. (2012) 33:141–9. 10.1007/s10059-012-2192-6 22286230 PMC3887716

[72] Kang SM Kim AD Heo SJ Kim KN Lee SH Ko SC. Induction of apoptosis by diphlorethohydroxycarmalol isolated from brown alga, Ishige okamurae. J Funct Foods. (2012) 4:433–9. 10.1016/j.jff.2012.02.001

[73] Jina R Hye-Jung H In-Hye K Taek-Jeong N. Mechanism of inhibition of HepG2 cell proliferation by a glycoprotein from Hizikia fusiformis. Kor J Fish Aquat Sci. (2012) 45:553–60. 10.5657/KFAS.2012.0553

[74] Yang L Wang P Wang H Li Q Teng H Liu Z. Fucoidan derived from Undaria pinnatifida induces apoptosis in human hepatocellular carcinoma SMMC-7721 cells *via* the ROS-mediated mitochondrial pathway. Mar Drugs. (2013) 11:1961–76. 10.3390/md11061961 23752353 PMC3721216

[75] Terasaki M Murase W Kamakura Y Kawakami S Kubota A Kojima H. A biscuit containing fucoxanthin prevents colorectal carcinogenesis in mice. Nutr Cancer. (2022) 74:3651–61. 10.1080/01635581.2022.2086703 35695489

[76] Nguyen VT. Ji Qian Z Lee B Heo SJ Kim KN Jeon YJ. Fucoxanthin derivatives from Sargassum siliquastrum inhibit matrix metalloproteinases by suppressing NF-kB and MAPKs in human fibrosarcoma cells. Algae. (2014) 29:355–66. 10.4490/algae.2014.29.4.355

[77] Kim KS Cui X Lee DS Sohn JH Yim JH Kim YC. Anti-inflammatory effect of neoechinulin a from the marine fungus Eurotium sp. SF-5989 through the suppression of NF-κB and p38 MAPK Pathways in lipopolysaccharide-stimulated RAW264.7 macrophages. Molecules. (2013) 18:13245–59. 10.3390/molecules181113245 24165583 PMC6270177

[78] Hsu HC Chen MH Yeh ML Chen WJ. Antibacterial and anticancer activities of pleurocidin-amide, a potent marine antimicrobial peptide derived from winter flounder, pleuronectes americanus. Mar Drugs. (2022) 20:519. 10.3390/md20080519 36005521 PMC9409841

[79] Liu J Ma L Wu N Liu G Zheng L Lin X. Aplysin sensitizes cancer cells to TRAIL by suppressing P38 MAPK/survivin pathway. Mar Drugs. (2014) 12:5072–88. 10.3390/md12095072 25257790 PMC4178493

[80] Nazari M Serrill JD Wan X Nguyen MH Anklin C Gallegos DA. New mandelalides expand a macrolide series of mitochondrial inhibitors. J Med Chem. (2017) 60:7850–62. 10.1021/acs.jmedchem.7b00990 28841379 PMC5702619

[81] Hernández-Zazueta MS García-Romo JS Noguera-Artiaga L Luzardo-Ocampo I Carbonell-Barrachina ÁA Taboada-Antelo P. Octopus vulgaris ink extracts exhibit antioxidant, antimutagenic, cytoprotective, antiproliferative, and proapoptotic effects in selected human cancer cell lines. J Food Sci. (2021) 86:587–601. 10.1111/1750-3841.15591 33462812

[82] Khalifa SAM Elias N Farag MA Chen L Saeed A Hegazy MF. Marine natural products: a source of novel anticancer drugs. Mar Drugs. (2019) 17:491. 10.3390/md17090491 31443597 PMC6780632

[83] Lin Y Qi X Liu H Xue K Xu S Tian Z. The anti-cancer effects of fucoidan: a review of both *in vivo* and *in vitro* investigations. Cancer Cell Int. (2020) 20:154. 10.1186/s12935-020-01233-8 32410882 PMC7206694

[84] Alekseyenko TV Zhanayeva SY Venediktova AA Zvyagintseva TN Kuznetsova TA Besednova NN. Antitumor and antimetastatic activity of fucoidan, a sulfated polysaccharide isolated from the Okhotsk Sea Fucus evanescens brown alga. Bull Exp Biol Med. (2007) 143:730–2. 10.1007/s10517-007-0226-4 18239813

[85] Eid SY El-Readi MZ Wink M. Carotenoids reverse multidrug resistance in cancer cells by interfering with ABC-transporters. Phytomedicine. (2012) 19:977–87. 10.1016/j.phymed.2012.05.010 22770743

[86] Kumar SR Hosokawa M Miyashita K. Fucoxanthin: a marine carotenoid exerting anti-cancer effects by affecting multiple mechanisms. Mar Drugs. (2013) 11:5130–47. 10.3390/md11125130 24351910 PMC3877908

[87] Sanjeewa KK Fernando IP Kim EA Ahn G Jee Y Jeon YJ. Anti-inflammatory activity of a sulfated polysaccharide isolated from an enzymatic digest of brown seaweed Sargassum horneri in RAW 2647 cells. Nutr Res Pract. (2017) 11:3–10. 10.4162/nrp.2017.11.1.3 28194259 PMC5300944

[88] Echave J Otero P Garcia-Oliveira P Munekata PES Pateiro M Lorenzo JM. Seaweed-derived proteins and peptides: promising marine bioactives. Antioxidants. (2022) 11:176. 10.3390/antiox11010176 35052680 PMC8773382

[89] Rauf A Khalil AA Khan M Anwar S Alamri A Alqarni AM. Can be marine bioactive peptides (MBAs) lead the future of foodomics for human health? Crit Rev Food Sci Nutr. (2022) 62:7072–116. 10.1080/10408398.2021.1910482 33840324

[90] Aguilar-Briseno JA Cruz-Suarez LE Sassi JF Ricque-Marie D Zapata-Benavides P Mendoza-Gamboa E. Sulphated polysaccharides from Ulva clathrata and Cladosiphon okamuranus seaweeds both inhibit viral attachment/entry and cell-cell fusion, in NDV infection. Mar Drugs. (2015) 13:697–712. 10.3390/md13020697 25629385 PMC4344596

[91] Wang S Zhu F. Dietary antioxidant synergy in chemical and biological systems. Crit Rev Food Sci Nutr. (2017) 57:2343–57. 10.1080/10408398.2015.1046546 26176981

[92] Sandner G Konig A Wallner M Weghuber J. Alternative model organisms for toxicological fingerprinting of relevant parameters in food and nutrition. Crit Rev Food Sci Nutr. (2022) 62:5965–82. 10.1080/10408398.2021.1895060 33683153

[93] Frontela-Saseta C Lopez-Nicolas R Gonzalez-Bermudez CA Martinez-Gracia C Ros-Berruezo G. Anti-inflammatory properties of fruit juices enriched with pine bark extract in an in vitro model of inflamed human intestinal epithelium: the effect of gastrointestinal digestion. Food Chem Toxicol. (2013) 53:94–9. 10.1016/j.fct.2012.11.024 23220608

[94] Kaulmann A Andre CM Schneider YJ Hoffmann L Bohn T. Carotenoid and polyphenol bioaccessibility and cellular uptake from plum and cabbage varieties. Food Chem. (2016) 197(Pt A):325–32. 10.1016/j.foodchem.2015.10.049 26616956

[95] Ponce de. Leon-Rodriguez MDC, Guyot JP, Laurent-Babot C. Intestinal in vitro cell culture models and their potential to study the effect of food components on intestinal inflammation. Crit Rev Food Sci Nutr. (2019) 59:3648–66. 10.1080/10408398.2018.1506734 30277794

